# Gomisin N Improves Insulin Secretion by Alleviating Endoplasmic Reticulum and Oxidative Stress in Pancreatic Beta Cells

**DOI:** 10.1155/ije/9046981

**Published:** 2026-08-08

**Authors:** Joo Young Lee, Chong Kun Cheon

**Affiliations:** ^1^ Department of Pediatrics, Pusan National University School of Medicine, Pusan National University Children’s Hospital, Yangsan 50612, Gyeongsangnam-do, South Korea, pnuyh.or.kr; ^2^ Research Institute for Convergence of Biomedical Science and Technology, Pusan National University Yangsan Hospital, Yangsan 50612, Gyeongsangnam-do, South Korea, pnuyh.or.kr

**Keywords:** antioxidant activity, ER stress, gomisin N, oxidative stress

## Abstract

This study investigated the effects of gomisin N (25 μM) using a cyclopiazonic acid (CPA, 50 μM)–induced injury model of MIN6 pancreatic beta‐cells. The results demonstrated that gomisin N effectively reduced the expression of endoplasmic reticulum (ER) stress markers that were increased by CPA. Furthermore, gomisin N treatment improved insulin secretion from MIN6 cells, which had been reduced by CPA. These findings suggest that alleviation of ER stress and oxidative stress ultimately supports the correct folding and maturation of insulin precursors and enhances beta‐cell viability, leading to functional normalization. Gomisin N exhibited antioxidant effects by significantly reducing reactive oxygen species (ROS) levels that were increased by CPA. Given that ER stress and oxidative stress are closely linked and cooperatively drive cellular damage, the dual stress‐relieving properties of gomisin N are critical for beta‐cell protection. By alleviating ER stress and reducing oxidative stress, gomisin N acts to protect and restore insulin secretory function in beta‐cells. Therefore, gomisin N could play a beneficial role in protecting and improving pancreatic beta‐cell function, particularly under stress conditions.

## 1. Introduction

Diabetes is a chronic metabolic disease with rapidly increasing prevalence worldwide, characterized by hyperglycemia and associated with various complications such as cardiovascular disease, kidney disease, and neuropathy [1–3]. The progressive loss of pancreatic beta‐cell mass and function is recognized as the central pathophysiological driver of the onset and progression of type 2 diabetes [4, 5]. Pancreatic beta‐cells are essential for maintaining blood glucose homeostasis by producing and secreting insulin in response to blood glucose levels [6]. Consequently, elucidating the cytopathological processes that govern pancreatic beta‐cell failure is paramount for developing targeted therapeutic interventions. At the cellular level, various metabolic and inflammatory stressors, such as chronic hyperglycemia and hyperlipidemia, can impose an excessive burden on the ER within pancreatic beta‐cells, leading to the development of ER stress [7, 8]. The ER is an organelle essential for various cellular functions, including protein synthesis, folding, modification, transport, and maintenance of calcium homeostasis [9]. When ER stress occurs due to the accumulation of misfolded proteins, the cell activates the unfolded protein response (UPR) to restore ER homeostasis [10]. While the UPR initially plays a protective role for the cell, prolonged and excessive ER stress can instead promote cell death, accelerating pancreatic beta‐cell dysfunction and loss [8, 10]. At the molecular scale, this pathogenic cascade is amplified by a deleterious crosstalk with the cellular oxidative environment. UPR activation is closely associated with the regulation of the oxidative environment within the ER [11]. ROS are inherently produced during physiological processes like protein disulfide bond formation within the ER, but under intensified ER stress, excessive ROS production leads to overwhelming oxidative stress [12]. These heightened ROS levels further damage critical proteins and lipids within the ER, thereby impairing ER function and establishing a vicious cycle that perpetuates and exacerbates ER stress [13]. Pancreatic beta‐cells are particularly vulnerable to oxidative stress owing to their relatively limited antioxidant capacity, which contributes significantly to impaired insulin production and secretion as well as cell death [14]. Therefore, effectively regulating both ER stress and oxidative stress simultaneously could be an important therapeutic strategy for preserving pancreatic beta‐cell function and inhibiting the progression of diabetes.

In this context, gomisin N is a lignan component derived from Schisandra chinensis, which has been actively studied for its pharmacological effects [15]. According to previous studies, gomisin N has been reported to exhibit various physiological activities, including potent antioxidant, anti‐inflammatory, anticancer, liver‐protective, and neuroprotective effects [15, 16]. For example, gomisin N improved lipid metabolism and attenuated oxidative stress in an alcohol‐induced liver damage model and demonstrated hepatoprotective effects in a nonalcoholic fatty liver disease (NAFLD) model by inhibiting ER stress [17]. Additionally, in neural cell models, it exhibited neuroprotective effects by counteracting oxidative stress, and it is also known to induce cell death in certain cancer cells [18, 19]. Notably, our research group previously confirmed that gomisin N ameliorates obesity by inhibiting ER stress in a *Drosophila melanogaster* obesity model [20]. Crucially, while gomisin N is known for its hepatoprotective, neuroprotective, and lipid‐lowering capabilities, no prior in vitro study has targeted pancreatic beta‐cells or explored its direct glucose‐lowering effects. To address this gap, the present study employed cyclopiazonic acid (CPA), an inhibitor of sarco/endoplasmic reticulum Ca^2+^‐ATPase (SERCA) that induces ER stress in pancreatic beta‐cells [21], to investigate whether gomisin N exerts protective effects against CPA‐induced beta‐cell injury and whether these effects are accompanied by improvements in insulin secretory function.

The present study aims to provide a novel mechanistic foundation for evaluating gomisin N as a potential therapeutic agent against pancreatic beta‐cell dysfunction in type 2 diabetes.

## 2. Materials and Methods

### 2.1. Chemicals

CPA (catalog no. HY‐N6686; MedChemExpress, Monmouth Junction, NJ, USA) and gomisin N (catalog no. CFN90125; ChemFaces, Wuhan, China) were used in this study. CPA and gomisin N were dissolved in dimethyl sulfoxide (DMSO) and stored at −20°C as stock solutions. Working concentrations were prepared by diluting stock solutions in cell culture medium immediately before use.

### 2.2. Cell Culture Conditions

The pancreatic beta cell line MIN6 (RRID: CVCL_0431) was used in this study. The cells originated from the pancreatic islets of a male Mus musculus. The cell line was obtained from Sigma‐Aldrich (St. Louis, Mo, USA; catalog no. SCC623) in 2022. According to the supplier, the cells were authenticated based on morphology and growth characteristics and are not listed as a misidentified or contaminated cell line in the International Cell Line Authentication Committee (ICLAC) database or Cellosaurus. Cells between passages 5–8 were used in this study. Mycoplasma testing was performed retrospectively in our laboratory using a PCR‐based assay on cultures derived from stored passage 5 cells, confirming that the cultures were mycoplasma negative. MIN6 cells were cultured in Dulbecco’s modified Eagle medium high glucose with sodium pyruvate medium containing 10% heat‐inactivated fetal bovine serum, 100 U/mL penicillin, and 100 µg/mL streptomycin in the presence of 5% CO_2_ at 37°C. MIN6 cells at 70% confluence were treated with 50 μM CPA alone or cotreated with 50 μM CPA and 25 μM gomisin N for 24 h. Cell viability assay for CPA and gomisin N is provided in Figure S1.

### 2.3. Real‐Time Quantitative PCR Analysis

After treatment with CPA and gomisin N for 24 h, real‐time quantitative polymerase chain reaction (qPCR) analysis was performed. Total RNA from MIN6 cells was extracted using TRIzol reagent (catalog no. 15596026; Invitrogen, Carlsbad, CA, USA). RNA was reverse transcribed using the M‐MLV cDNA Synthesis Kit (catalog no. EZ006S; Enzynomics, Daejeon, Korea). Gene expression was measured using TOPreal SYBR Green qPCR PreMix (catalog no. RT500M; Enzynomics, Daejeon, Korea). Primer sets used for amplification are listed in supporting Table 1. Relative gene expression levels were calculated by normalizing the transcript levels of the target genes to those of Rplp0.

### 2.4. Western Blot Analysis and Densitometry

For western blot analysis, cells were lysed with RIPA buffer (catalog no. BRA0500; BIOMAX, Guri, Korea) after phosphate‐buffered saline (PBS) rinsing, and protein concentrations were determined using the Pierce BCA Protein Assay Kit (catalog no. 23227; Thermo Fisher Scientific, Rockford, IL, USA). Equal amounts of protein (10 μg per lane) were separated by 10% sodium dodecyl sulfate–polyacrylamide gel electrophoresis (SDS‐PAGE) at 100 V and transferred to nitrocellulose membranes (Hybond ECL, Amersham) at 250 mA for 90 min. Membranes were blocked with 3% bovine serum albumin (BSA) in tris‐buffered saline with Tween‐20 (TBST) for 1 h at room temperature and then incubated overnight at 4°C with the following mouse monoclonal primary antibodies from Santa Cruz Biotechnology (Dallas, TX, USA): anti‐Grp94 (catalog no. SC‐393402), anti‐BiP/Grp78 (catalog no. SC‐166490), anti‐Atf4 (catalog no. SC‐39066), and anti‐*β*‐actin (catalog no. SC‐69879). After TBST washes, a horseradish peroxidase–conjugated mouse IgG kappa binding protein (m‐IgGκ BP‐HRP; catalog no. SC‐516102; Santa Cruz Biotechnology) was applied for 1 h at room temperature, followed by further TBST washes. Signals were developed using an enhanced chemiluminescence substrate (Amersham), captured on a luminescent image analyzer (ATTO, Tokyo, Japan), and quantified by densitometry in ImageJ (Fiji). For ImageJ analysis, band intensities were measured using the rectangular selection and gel analyzer tools with local background subtraction; values were normalized to the corresponding loading control and expressed relative to the control condition.

### 2.5. Insulin Secretion Analysis

MIN6 cells in 6‐well plates were incubated for 15 min in Krebs‐Ringer buffer (NaCl 130 mM, KCl 4.7 mM, NaH₂PO₄ 0.5 mM, MgSO₄ 1 mM, CaCl₂ 1.5 mM, 4‐(2‐hydroxyethyl)‐1‐piperazineethanesulfonic acid (HEPES) 2.4 mM, BSA 1 mg/mL, pH 7.4) with 2.8 mM glucose. Next, cells were stimulated by incubation in Krebs‐Ringer buffer containing 16.8 mM glucose for 15 min. At the end of the incubation period, the buffer from each well was collected for insulin quantification. Secreted insulin was measured using the Ultra‐Sensitive Mouse Insulin ELISA Kit (catalog no. 90080; Crystal Chem, Elk Grove Village, IL, USA) according to the manufacturer’s protocol.

### 2.6. Mitochondrial ROS Assay

Mitochondrial ROS levels were measured using MitoSOX Red (mitochondrial superoxide indicator; catalog no. M36008; Invitrogen, Carlsbad, CA, USA). After the treatments, MIN6 cells were washed three times with PBS and then incubated with 5 µm, MitoSOX Red for 30 min in a 37°C incubator under 5% CO_2_. Cells were then washed three times with PBS. Fluorescence images were acquired at an emission wavelength of 610 nm using a Nikon Eclipse E600 fluorescence microscope (Nikon, Tokyo, Japan) equipped with a rhodamine‐compatible filter set. All images were captured under strictly identical acquisition settings—including exposure time, light intensity, and objective magnification—to ensure intersample comparability. The red channel was extracted in ImageJ (Fiji) and converted to 16‐bit. Background was removed using a 50‐pixel rolling ball radius, and the image was slightly smoothed with a Gaussian blur filter. Images were converted to binary masks using automated intensity thresholding, followed by watershed segmentation to separate adjacent cells. Fluorescence quantification was performed using the Analyze Particles function; for each cell, the corrected total cell fluorescence (CTCF) was calculated as CTCF = integrated density − (area of selected cell × mean fluorescence of background readings).

### 2.7. Statistical Analysis

Data from the two experimental groups were compared using an unpaired two‐tailed Student’s *t*‐test. Multiple comparisons were performed using one‐way analysis of variance (ANOVA), followed by Tukey’s post hoc test. Significant differences were considered when *p* < 0.05.

## 3. Results

### 3.1. Gomisin N Attenuates CPA‐Induced Endoplasmic Reticulum Stress in Pancreatic Beta‐Cells

ER stress is a pathological condition that arises when the UPR, which is crucial for pancreatic beta‐cell homeostasis, is pushed beyond its adaptive limits. We evaluated the impact of gomisin N (Go) on ER stress induced by CPA in pancreatic beta‐cells.

Prior to the main experiments, we assessed the basal physiological safety of the treatment conditions and characterized the time‐dependent expression of ER stress markers. To this end, we analyzed the expression of activating transcription factor 4 (Atf4), a key transcriptional regulator of the PERK signaling pathway governing cell survival and apoptosis, and BiP/Grp78, a major ER chaperone involved in the processing of misfolded proteins, following gomisin N and CPA treatment. As a result, treatment with gomisin N alone for either 6 or 24 h did not induce aberrant expression of ER stress markers in MIN6 cells, demonstrating the favorable basal safety profile of the compound. In contrast, the CPA‐induced upregulation of BiP/Grp78 and Atf4 protein expression, as well as the protective effect of gomisin N in attenuating these responses, was most pronounced after 24 h of treatment (Figure S2A). Based on these validation results, all subsequent experiments were, therefore, performed using the 24‐h treatment protocol, except for the gomisin N‐alone treatment group.

**FIGURE 1:**
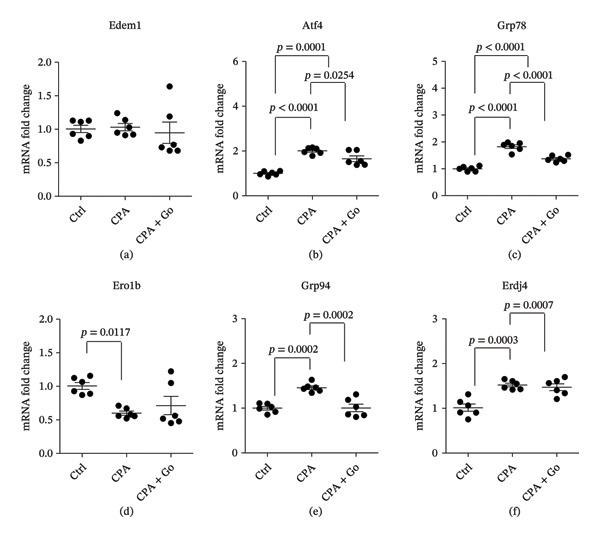
Effects of gomisin N (Go) on endoplasmic reticulum (ER) stress markers in pancreatic *β*‐cells. Mouse pancreatic *β*‐cell lines were treated with 50 μM CPA alone or cotreated with 50 μM CPA and 25 μM Go for 24 h. The mRNA expression levels of representative ER stress–related genes were quantified by real‐time quantitative PCR (A–F). Gene expression was normalized to the housekeeping gene Rplp0 to minimize interexperiment variability. The sample size was six (*n* = 6). Statistical significance was evaluated relative to the untreated control group, and *p* values are indicated.

**FIGURE 2:**
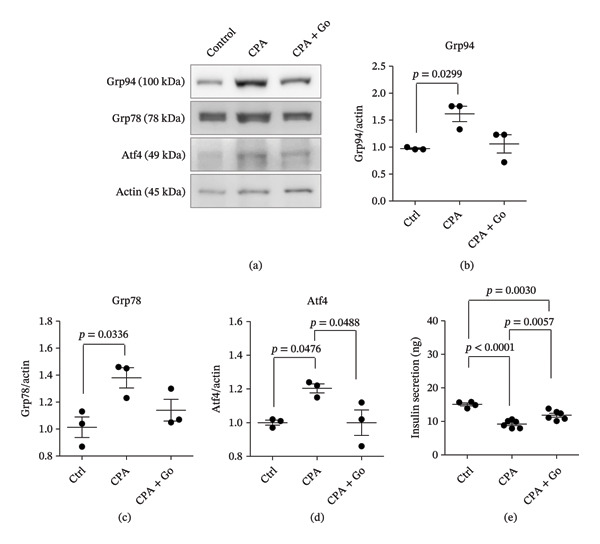
Gomisin N (Go) attenuates CPA‐induced ER stress responses and improves insulin secretion in pancreatic *β*‐cells. Mouse pancreatic *β*‐cell lines were treated with 50 μM CPA alone or cotreated with 50 μM CPA and 25 μM Go for 24 h. (A) Representative western blot images showing protein expression of the ER stress markers GRP94, BiP/GRP78, and ATF4. Actin served as a loading control. (B–D) Band intensities were quantified from three independent experiments using ImageJ, and the data are presented as scatter plots with mean ± SEM. (E) To evaluate *β*‐cell function, cells were first incubated in Krebs–Ringer buffer containing 2.8 mM glucose to determine basal insulin secretion, followed by stimulation with Krebs–Ringer buffer containing 16.8 mM glucose to induce insulin release. Secreted insulin in the culture medium was measured using a high‐sensitivity insulin ELISA kit. Data are presented as mean ± SEM from three independent experiments. Statistical significance was assessed, and *p* values are reported.

Using this validated experimental condition, we next investigated the effects of gomisin N on the PERK signaling pathway and ER chaperone expression. CPA treatment increased Atf4 mRNA and protein expression by more than 2.0‐fold and 1.5‐fold, respectively (Figures 1B and 2A,D). Cotreatment with gomisin N significantly attenuated the CPA‐induced upregulation of Atf4 at both the mRNA and protein levels (Figures 1B and 2D), indicating that gomisin N suppresses excessive activation of the PERK signaling pathway. Similarly, CPA markedly increased both the mRNA (Figure 1C) and protein (Figure 2A,C) expression of BiP/Grp78, whereas gomisin N cotreatment significantly reduced these elevations. Likewise, the expression of Grp94, another major ER chaperone, was significantly increased at both the mRNA (Figure 1E) and protein (Figure 2A,B) levels following CPA treatment, and this increase was significantly attenuated by gomisin N cotreatment. Uncropped, full‐length western blot images for these ER stress markers from three independent replicates are provided in Figure S3.

These findings indicate that gomisin N treatment ameliorates CPA‐induced ER stress and modulates the ER stress response, thereby enabling cells to better cope with excessive cellular stress.

### 3.2. Gomisin N Alleviates Pancreatic Beta‐Cell ER Stress and Improves Insulin Secretion

The primary function of pancreatic beta‐cells is to produce and secrete insulin in response to blood glucose levels. Reduced ER stress promotes the proper folding and maturation of proinsulin, which can ultimately lead to efficient insulin production and secretion. CPA induces ER stress through inhibition of SERCA, which in turn impairs insulin secretory function. Therefore, we investigated whether gomisin N could preserve insulin secretory function under ER stress conditions. Consistent with its lack of effect on basal ER stress marker expression, gomisin N alone did not alter basal insulin secretion (Figure S2B). On the other hand, as shown in Figure 2E, the CPA‐treated group exhibited a marked reduction in insulin secretion in response to high glucose stimulation compared with the control, indicating impaired insulin secretory function. Notably, the group treated with gomisin N alongside CPA showed a significant recovery of insulin secretion that was previously compromised by CPA, approaching the levels observed in the control group. These findings indicate that gomisin N can improve pancreatic beta‐cell function, in part by reducing the expression of ER stress markers such as BiP/Grp78, Grp94, and Atf4.

### 3.3. Gomisin N Reduces Oxidative Stress Induced by CPA

**FIGURE 3:**
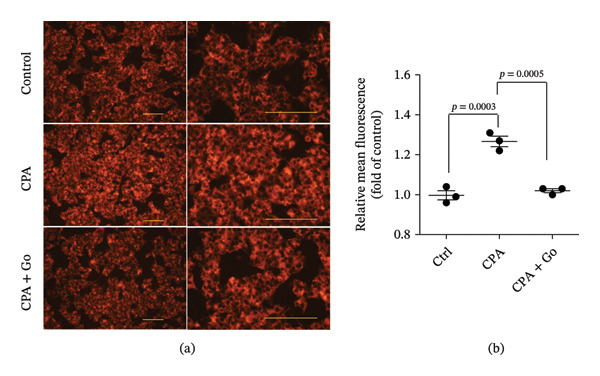
Antioxidant effects of gomisin N (Go) on mitochondrial superoxide in pancreatic *β*‐cells. (A) Mitochondrial superoxide levels were evaluated by staining MIN6 cells with MitoSOX Red (5 µM). Fluorescence images were acquired at an emission wavelength of 610 nm using identical exposure times and light intensity settings for all samples to ensure intersample comparability. Representative fluorescence images are shown; scale bar = 200 μM. (B) Quantification of MitoSOX red fluorescence intensity was performed using ImageJ (Fiji), and results were expressed as fold of control. Data are presented as mean ± SEM from four independent experiments. Statistical significance was determined by one‐way ANOVA followed by Tukey’s post hoc test.

ER stress is a major contributor to increased reactive oxygen species (ROS) within cells, and these two phenomena interact closely to form a vicious cycle leading to cellular damage. We measured mitochondrial ROS levels using a mitochondrial superoxide indicator (MitoSOX Red) (Figure 3). Visually, the CPA‐treated group exhibited a clear increase in red fluorescence intensity compared with the control group, indicating elevated mitochondrial ROS levels (Figure 3A). In the gomisin N cotreated group, the increased red fluorescence intensity induced by CPA was noticeably reduced.

Quantitative analysis further confirmed that mitochondrial ROS levels were significantly elevated by approximately 1.2‐fold in the CPA‐treated group compared with the control (Figure 3B). However, gomisin N cotreatment significantly mitigated these elevated ROS levels, bringing them closer to the control levels. Raw fluorescence images corresponding to these results are provided in Figure S4.

These results suggest that the antioxidant action of gomisin N is associated with reduced CPA‐induced mitochondrial ROS and may contribute to the alleviation of ER stress and the enhancement of insulin secretion in pancreatic beta‐cells.

## 4. Discussion

Our findings demonstrate that gomisin N effectively mitigates ER stress in pancreatic beta‐cells by modulating key components of the UPR. The observed reduction in the expression of Atf4, BiP/Grp78, and Grp94 suggests that gomisin N influences critical molecular pathways of the UPR [9]. Specifically, it is associated with reduced expression of both the initial compensatory chaperones, reflected by upregulation of BiP/Grp78 and Grp94, and the downstream effector Atf4. This combined effect is likely to help restore ER homeostasis and prevent overactivation of the stress response.

The decreased Atf4 expression is particularly significant. Atf4 plays a crucial role in promoting the expression of proapoptotic genes during prolonged and severe ER stress [22]. Therefore, the ability of gomisin N to reduce Atf4 suggests a potential mechanism for enhancing cell survival by attenuating apoptosis‐related pathways in beta‐cells. Protecting beta‐cell viability is a paramount goal in diabetes management, highlighting the therapeutic relevance of gomisin N.

Furthermore, the normalization of BiP/Grp78 and Grp94 expression is indicative of an improved ER protein‐folding environment. These chaperones are essential for proper protein folding, and their overexpression signals a heavy protein load and the accumulation of misfolded proteins within the ER [9]. By returning their expression to normal levels, gomisin N could positively impact the correct folding and maturation of insulin precursor proteins, ultimately supporting normal insulin production and secretion. This link between ER stress reduction and improved cellular function is supported by our observation that gomisin N significantly enhanced insulin secretion, counteracting the impairment caused by CPA‐induced ER stress. The reduction in BiP/Grp78 and Grp94 observed in this study reflects a broader restoration of ER proteostasis that extends beyond the PERK/Atf4 axis alone. Under normal conditions, BiP/Grp78 binds and inactivates the transmembrane ER stress sensors IRE1 and Atf6. Once liberated, IRE1 drives XBP1 mRNA splicing to promote ER‐associated degradation (ERAD), while Atf6 translocates to the Golgi for proteolytic cleavage, subsequently entering the nucleus to upregulate BiP/Grp78 and Grp94 expression [23]. Although this study primarily focused on the PERK‐Atf4 axis, the prominent downregulation of BiP/Grp78 and Grp94 by gomisin N strongly implies a coordinated suppression of the interconnected IRE1 and Atf6 pathways. It is highly plausible that gomisin N alleviates the overall ER stress burden, allowing BiP/Grp78 to rebind and silence these sensors, thereby preventing prolonged UPR‐mediated beta‐cell apoptosis [24]. While the direct alterations in the IRE1 and Atf6 branches remain to be quantified, these mechanisms represent promising directions for future research to fully map the therapeutic network of gomisin N.

The implications for diabetes, particularly type 2 diabetes, are substantial. A hallmark of this disease is beta‐cell dysfunction and impaired insulin secretion, alongside insulin resistance [25]. The findings of this study, demonstrating the ability of gomisin N to improve impaired insulin secretory function under ER stress, suggest its potential as a novel therapeutic candidate for blood glucose control. Unlike many existing diabetes medications that primarily improve insulin sensitivity or directly stimulate insulin secretion [26], gomisin N supports beta‐cell function and augments insulin secretion by alleviating ER stress. This mechanism of action positions gomisin N as a promising lead compound for developing new diabetes treatments.

Beyond its effects on ER stress, gomisin N also addresses the interconnected issue of oxidative stress. ER stress and oxidative stress are not merely co‐occurring phenomena but are intimately linked, reciprocally exacerbating each other and leading to cell damage and dysfunction [9].

Our data demonstrate that gomisin N effectively reduces CPA‐induced mitochondrial ROS in beta‐cells, while concurrently modulating the expression of core ER stress markers, including Atf4, BiP/Grp78, and Grp94. This antioxidant activity, integrated with direct ER stress relief, may be one of the key mechanisms through which gomisin N contributes to the overall alleviation of cellular stress and the enhancement of insulin secretion. Notably, this dual‐action profile appears to represent a mechanistic advantage over other major Schisandra chinensis lignans. Schisandrin B has been reported to exert antidiabetic effects primarily through GLP‐1 receptor‐mediated stimulation of insulin secretion [27], while the cytoprotective properties of schisandrin A and B have been attributed largely to antioxidant and anti‐inflammatory pathways in nonpancreatic cell types [28]. Importantly, direct suppression of the ER stress chaperone induction cascade—specifically GRP78, GRP94, and the downstream PERK–ATF4 axis—within beta‐cells has not been reported for either congener. In this regard, gomisin N’s simultaneous attenuation of mitochondrial ROS and ER stress markers in beta‐cells may provide a more comprehensive and complementary protective mechanism against the reciprocal cellular stresses that drive beta‐cell dysfunction in diabetes.

Nevertheless, the present study has certain inherent limitations that merit consideration. This investigation relied exclusively on the in vitro MIN6 pancreatic beta‐cell monolayer model, and in vivo validation using primary pancreatic islets or diabetic animal models was not performed in this initial phase. In vivo diabetic models involve complex systemic interactions across multiple organs—including the liver, adipose tissue, and skeletal muscle—which can confound the interpretation of direct, cell‐autonomous effects on beta‐cells. By employing a targeted in vitro approach, we successfully isolated and characterized the beta‐cell‐specific protective mechanisms of gomisin N against CPA‐induced ER and oxidative stress without the interference of systemic metabolic variables. Nonetheless, we acknowledge that this monolayer model cannot fully recapitulate in vivo drug pharmacokinetics, bioavailability, or the complex pancreatic microenvironment, including islet paracrine signaling and the influence of the surrounding vasculature.

In conclusion, our findings demonstrate that gomisin N restores insulin secretory function by synergistically alleviating endoplasmic reticulum and oxidative stress. These protective effects position gomisin N as a potential lead natural compound for type 2 diabetes. Moving forward, further in vivo studies are warranted to rigorously evaluate its pharmacodynamics, systemic bioavailability, and ultimate clinical translational potential.

## Funding

This work was supported by the Korean Society of Pediatric Endocrinology Grant (2016‐03).

## Conflicts of Interest

The authors declare no conflicts of interest.

## Supporting Information

Additional supporting information can be found online in the Supporting Information section.

## Supporting information


**Supporting Information** The following supporting information is available: Figure S1. Cell viability analysis for CPA and gomisin N. Cells were treated with various concentrations of CPA (A) and gomisin N (B) for 24 h. Cell viability was assessed by measuring intracellular ATP levels as an indicator of metabolic activity and cell health. ATP levels were quantified using a luminescence‐based assay, and data are presented as a percentage relative to untreated control cells. Figure S2. Validation of the basal safety of gomisin N and time‐dependent attenuation of CPA‐induced ER stress. MIN6 cells were treated with CPA and/or Go for 6 or 24 h. (A) Representative western blot of Grp78 and Atf4. *β*‐Actin was used as the loading control. (B) Glucose‐stimulated insulin secretion in MIN6 cells treated with vehicle (Ctrl) or Go. Data are presented as mean ± SD (*n* = 3). No significant difference was observed between groups (Student’s *t*‐test, *p* > 0.05). Figure S3. Uncropped western blot images of ER stress markers from three independent experiments. Full‐length western blots show protein expression of GRP94, BiP/GRP78, and ATF4 from three independent experiments corresponding to Figure 2A. Actin served as the loading control. Band intensities from these blots were quantified using ImageJ to generate the data shown in Figure 2B–D. Figure S4. Raw fluorescence images of mitochondrial ROS production assessed by MitoSOX Red staining. Representative fluorescence images of pancreatic *β*‐cells stained with MitoSOX Red are shown from three independent experiments (labeled 1–3). These raw images show mitochondrial ROS levels in control, CPA, and CPA + Go groups and were used for the quantification presented in Figure 3B. Table S1. Primer sequences used for quantitative real‐time PCR.

## Data Availability

The data that support the findings of this study are available from the corresponding author upon reasonable request.
