# Supplementary material for: Gomisin N Improves Insulin Secretion by Alleviating Endoplasmic Reticulum and Oxidative Stress in Pancreatic Beta Cells
Source: Int J Endocrinol. 2026 Aug 8;2026:9046981. doi: 10.1155/ije/9046981 (PMC13451963; doi:10.1155/ije/9046981)
Supplement: Supplementary file 1 [file IJE-2026-9046981-s001.docx]

**Figure-S1**

**Figure S1.** Cell viability analysis for CPA and gomisin N. Cells were treated with various concentrations of CPA (A) and gomisin N (B) for 24 hours. Cell viability was assessed by measuring intracellular ATP levels as an indicator of metabolic activity and cell health. ATP levels were quantified using a luminescence-based assay, and data are presented as a percentage relative to untreated control cells.

**Figure-S2**

**Figure S2.** Validation of the basal safety of gomisin N and time-dependent attenuation of CPA-induced ER stress. MIN6 cells were treated with CPA and/or Go for 6 or 24 h. (A) Representative Western blot of Grp78 and Atf4. β-Actin was used as the loading control. (B) Glucose-stimulated insulin secretion in MIN6 cells treated with vehicle (Ctrl) or Go. Data are presented as mean ± SD (n = 3). No significant difference was observed between groups (Student's t-test, p > 0.05).

**Figure-S3**

**Figure S3.** Uncropped Western blot images of ER stress markers from three independent experiments. Full-length Western blots show protein expression of GRP94, BiP/GRP78, and ATF4 from three independent experiments corresponding to Figure 2A. Actin served as the loading control. Band intensities from these blots were quantified using ImageJ to generate the data shown in Figures 2B–D.

**Figure-S4**

**Figure S4.** Raw fluorescence images of mitochondrial ROS production assessed by MitoSOX^TM^ Red staining. Representative fluorescence images of pancreatic β-cells stained with MitoSOX™ Red are shown from three independent experiments (labeled 1–3). These raw images show mitochondrial ROS levels in Control, CPA, and CPA + Go groups and were used for the quantification presented in Figure 3B.

**Table S1.** Primer sequences used for quantitative real‑time PCR.

| **Gene symbol** | | **Oligo Seq (5`->3`)** | **Accession** |
| --- | --- | --- | --- |
| *Rplp0* | Forward | TGACATCGTCTTTAAACCCCG | NM_007475.5 |
|  | Reverse | TGTCTGCTCCCACAATGAAG |  |
| *Atf4* | Forward | GGTTCTCCAGCGACAAGG | NM_009716.3 |
|  | Reverse | GCATCGAAGTCAAACTCTTTCAG |  |
| *Bip* | Forward | AGTTGATATTGGAGGTGGGC | NM_001163434.1 |
|  | Reverse | CATTGAAGTAAGCTGGTACAGTAAC |  |
| *Ero1b* | Forward | CGTCCTTTAAATCCTTTGGCG | NM_001418471.1 |
|  | Reverse | ACACAAACCTTCTAGCCACG |  |
| *Grp94* | Forward | AACCTCTGCTCAACTGGATG | NM_011631.1 |
|  | Reverse | TTGTGCCTTCATGATCCTCTC |  |
| *Erdj4* | Forward | AAATAAAAGCCCTGATGCTGAAG | NM_013760 |
|  | Reverse | CCTCTTTGTCCTTTGCCATTG |  |
| *Edem1* | Forward | CAATGAAGGAGAAGGAGACCC | NM_138677 |
|  | Reverse | GCATCTTCCACATCCCCTATC |  |
